# The Regulatory RNA *ern0160* Confers a Potential Selective Advantage to *Enterococcus faecium* for Intestinal Colonization

**DOI:** 10.3389/fmicb.2021.757227

**Published:** 2021-11-10

**Authors:** Sophie Reissier, Killian Le Neindre, Valérie Bordeau, Loren Dejoies, Audrey Le Bot, Brice Felden, Vincent Cattoir, Matthieu Revest

**Affiliations:** ^1^Unité Inserm U1230, Université de Rennes 1, Rennes, France; ^2^Service de Bactériologie-Hygiène Hospitalière & CNR de la Résistance aux Antibiotiques (Laboratoire Associé ‘Entérocoques’), CHU de Rennes, Rennes, France; ^3^Service de Maladies Infectieuses et Réanimation Médicale, CHU de Rennes, Rennes, France

**Keywords:** *E. faecium*, VRE, sRNA, pathogenicity, animal model, gut colonization

## Abstract

The aim of this study was to evaluate the role of the regulatory small RNA (sRNA) Ern0160 in gastrointestinal tract (GIT) colonization by *Enterococcus faecium*. For this purpose, four strains of *E. faecium* were used, Aus0004 (WT), an *ern0160*-deleted Aus0004 mutant (Δ0160), a *trans*-complemented Δ0160 strain overexpressing *ern0160* (Δ0160_0160), and a strain Δ0160 with an empty pAT29 vector (Δ0160_pAT29). Strains were studied both *in vitro* and *in vivo*, alone and in competitive assays. In *in vitro* experiments, no difference was observed between WT and Δ0160 strains cultured single while Δ0160_0160 strain grew more slowly than Δ0160_pAT29. In competitive assays, the WT strain was predominant compared to the deleted strain Δ0160 at the end of the experiment. Then, *in vivo* experiments were performed using a GIT colonization mouse model. Several existing models of GIT colonization were compared while a novel one, combining ceftriaxone and amoxicillin, was developed. A GIT colonization was performed with each strain alone, and no significant difference was noticed. By contrast, significant results were obtained with co-colonization experiments. With WT + Δ0160 suspension, a significant advantage for the WT strain was observed from day 5 to the end of the protocol, suggesting the involvement of *ern*0160 in GIT colonization. With Δ0160_0160 + Δ0160_pAT29 suspension, the strain with the empty vector took the advantage from day 3 to the end of the protocol, suggesting a deleterious effect of *ern0160* overexpression. Altogether, these findings demonstrate the potential implication of Ern0160 in GIT colonization of *E. faecium*. Further investigations are needed for the identification of sRNA target(s) in order to decipher underlying molecular mechanisms.

## Introduction

Enterococci are ubiquitous Gram-positive cocci that are normal inhabitants of the human gut microbiota. *Enterococcus faecium*, which represents 15–25% of enterococcal isolates responsible for human infections, has been increasingly involved in healthcare-associated infections and hospital outbreaks (Goh et al., 2017; Weiner-Lastinger et al., 2020). Glycopeptides are usually used to treat serious *E. faecium* infections, but many clinical isolates have become resistant to vancomycin (VREF) especially in North America. In 2010, up to 80% of VREF were reported in the United States (O’Driscoll and Crank, 2015). According to the European Antimicrobial Resistance Surveillance Network (EARS-Net), the mean proportion of VREF among invasive isolates increased from 10.4 to 17.3% in European countries between 2014 and 2018 (European Centre for Disease Prevention and Control, 2019). These high rates of VREF prevalence could be attributable to the worldwide dissemination of a subpopulation of *E. faecium* hospital-adapted clones that belongs to the clonal complex 17 (CC17) and represents a public health concern (Cattoir and Giard, 2014). This CC17 actually corresponds to the phylogenetic clade A1 composed by the majority of strains responsible for infections and hospital outbreaks. Two other clades also exist, clade A2 that includes animal strains and sporadic human infection isolates and clade B that comprises human commensal fecal strains (Lebreton et al., 2013).

In recent years, bacterial regulatory RNAs, referred to as small RNAs (sRNAs), have been described as having a major role in various adaptive responses, including stress response, virulence, and antimicrobial resistance (Mraheil et al., 2010; Wagner and Romby, 2015; Klein and Raina, 2017). For example, SprX contributes to vancomycin resistance in *Staphylococcus aureus* (Eyraud et al., 2014) and some sRNAs are involved in stress response in *Enterococcus faecalis* (Michaux et al., 2014). Recently, 61 sRNA candidates were identified in *E. faecium* but there are currently no published data about their function (Sinel et al., 2017). Among them, 10 have been experimentally validated and their expression under sub-inhibitory concentrations (SICs) of daptomycin was observed. sRNA_0160 (renamed here Ern0160) appeared to be highly expressed and its expression decreased significantly under daptomycin exposure. Furthermore, *ern0160* is conserved in all studied strains of *E. faecium* belonging to the three different clades (Sinel et al., 2017). These initial positive results led us to study this sRNA to evaluate its potential role in intestinal colonization.

Because the gastrointestinal tract (GIT) serves as a major reservoir from which VREF can spread to the hospital environment and GIT colonization precedes infection, understanding GIT colonization mechanisms appears to be essential to better manage those infections and to limit VREF hospital spread (Donskey et al., 2000, 2001; Ubeda et al., 2010). *In vivo* GIT colonization experimental models with enterococci have been described for a long time, with about one-third of these studies exploring *E. faecium*. Most of them used mouse models and different antibiotic protocols to eliminate the animal commensal microbiota allowing to the establishment of colonization by the studied bacteria. Several different protocols for GIT colonization with enterococci have been published, and there is no consensus on molecules used as well as routes and durations of administration. For example, Heikens et al. (2009) used a protocol with subcutaneous (SC) ceftriaxone alone, administered for 12 days. Zhang et al. (2013) associated SC ceftriaxone 2 days before inoculation, with cefoxitin added to drinking water (DW) during the protocol. Montealegre et al. (2016) published in 2016 a model of *E. faecium* GIT colonization using a combination of SC clindamycin and gentamicin in DW only 4 days before inoculation. As these protocols are very different, it seemed interesting to compare their performances. Moreover, as VREF intestinal colonization mostly occurs in patients treated with broad-spectrum antibiotics, it seems important to use antibiotics frequently prescribed in clinics, such as third-generation cephalosporins or ampicillin, to study the mechanisms of VREF colonization (Dubin and Pamer, 2014).

The aim of this study was to evaluate the role of Ern0160 in *E. faecium* GIT colonization. For this purpose, *in vitro* experiments were performed and several existing models of GIT colonization were compared, while a novel one, as close as possible to human conditions, was developed for *in vivo* studies.

## Materials and Methods

### Bacterial Strains

Data regarding the strain characteristics are summarized in Table 1. The *E. faecium* Aus0004 wild-type (WT) reference strain was used (Lam et al., 2012). This vancomycin-resistant (*vanB*-positive) clinical isolate was recovered from a bacteremic patient and belongs to the CC17. The MIC of amoxicillin for Aus0004 strain was 256 mg/l.

**TABLE 1:** Bacterial strains and plasmids used in the study.

**Strains or plasmids**	**Relevant characteristics**	**References**
**Strains**		
*Enterococcus faecium*		
Aus0004 WT	*vanB*-positive *E. faecium* Aus0004 reference strain	Lam et al., 2012
Δ0160	*ern0160*-deleted Aus0004	This study
Δ0160_pAT29	Δ0160 carrying empty pAT29 vector	This study
Δ0160_Δ0160	Δ0160 carrying recombinant plasmid pAT29Ω0160	This study
*Escherichia coli*		
EC1000	Strain using for cloning	Leenhouts et al., 1996
**Plasmids**		
pWS3	Temperature-sensitive pG(+)host9-derived shuttle vector used for gene disruption (Spc^r^)	Zhang et al., 2011
pAT29	High-copy-number shuttle vector used for cloning (Spc^r^)	Trieu-Cuot et al., 1990
pAT29Ωern0160	Recombinant pAT29 plasmid containing *ern0160* with its native promoter and rho-independent terminator (Spc^r^)	This study

*Spc^*r*^, spectinomycin resistance.*

Three mutants were constructed to evaluate the role of Ern0160 in GIT colonization. As described by Zhang et al. (2011), an *ern0160*-deleted *E. faecium* Aus0004 mutant (named Δ0160) was constructed by allelic exchange with a truncated copy of the gene corresponding to 5′-3′ positions of *ern0160* using pWS3 and specific primers (Supplementary Table 1). The *ern0160* gene with its native promoter and rho-independent terminator was cloned into the pAT29 shuttle vector using specific primers (Trieu-Cuot et al., 1990). The recombinant plasmid was introduced into *Escherichia coli* EC1000 and then into *E. faecium* Δ0160 (Leenhouts et al., 1996). The *trans*-complemented strain overexpressing *ern0160* was named Δ0160_0160. A strain Δ0160 with an empty pAT29 vector was used as control (Δ0160_pAT29). The transformants were selected on media containing 100 mg/l (*E. coli*) or 300 mg/l (*E. faecium*) of spectinomycin.

### Bacterial Growth Curves and Competitive Assays

Growth curves were performed *in vitro* for each strain and WT was compared to Δ0160, while Δ0160_0160 was compared to Δ0160_pAT29. Strains were cultured aerobically overnight on Trypticase Soy (TS) agar (Thermo Fisher Scientific, Waltham, MA, United States) at 35 ± 2°C. A colony was cultured in 10 ml of Brain Heart Infusion (BHI) broth (Thermo Fisher Scientific, Waltham, MA, United States) for 18 h at 35 ± 2°C under ambient air. Bacterial cultures were then adjusted to DO_600_ 0.1, and the bacterial growth was evaluated by DO_600_ at each time point (2, 3, 4, 5, 6, 9, and 24 h). Two competitive assays were also performed, WT versus Δ0160 and Δ0160_0160 versus Δ0160_pAT29. Strains were initially mixed at a ratio of 1:1, and bacterial growth was measured in the same way as described above. Spectinomycin was added (300 mg/l) to BHI and TS media (BHIspec and TSspec) for all experiments with Δ0160_pAT29 and Δ0160_0160 strains to ensure the maintenance of the plasmid. For competitive assays, a sample of culture was plated on TS and TSSpec agar at each point of time, and each strain was identified by PCR, directly from colonies, under standard conditions and using specific primers (Supplementary Table 1). For each time point, 28 colonies were tested, and each experiment was performed independently three times.

### Mouse Model of Gastrointestinal Tract Colonization

The same strains used for *in vitro* experiments were studied in *in vivo* protocols. For all the experiments, bacterial suspensions were calibrated as follows to inoculate mice with 10^8^ cfu/ml. Each strain was grown aerobically overnight on TS agar at 35 ± 2°C. A colony was cultured in 10 ml of BHI broth for 18 h at 35 ± 2°C under ambient air. After centrifugation (15 min, 3,500 rpm), the pellet was resuspended in 10 ml of 0.9% saline and centrifuged again. This second pellet was resuspended in 5 ml of 0.9% saline, and the inoculum was quantified by serial dilutions plated on TS agar. For co-colonization experiment, a suspension of each strain was prepared as described above, adjusted to the same OD_600_, and mixed in a 1:1 ratio before being administered to mice. As previously, BHIspec and TSspec were used for all experiments with Δ0160_pAT29 and Δ0160_0160 strains. To ensure that mice GIT do not contain any *E. faecium*, an aerobic GIT microbiota study was performed on three cages of five mice. Ten fresh fecal pellets per cage, obtained by light abdominal massage, were suspended in 3 ml of 0.9% NaCl. This suspension was serially diluted and cultured on TS, BHI, and Bile Esculin Azide (BEA) agar plates (Sigma-Aldrich, Saint-Louis, MO, United States) aerobically at 35+/− 2°C for 24 h. The different colonies were then quantified and identified by MALDI-TOF mass spectrometry (Microflex, Bruker Daltonics, Billerica, MA, United States).

Six-week-old non-inbred female, specific pathogen-free, Swiss mice were purchased (Janvier Labs, Le Genest-Saint-Isle, France). Five animals were housed per cage, with controlled room temperature, a 12-h light–dark cycle, and sterile standard rodent food and water ad libitum. Mice were acclimated for 1 week prior to the experiment. The experimental protocol was in keeping with French legislation on animal experimentation and approved by the Adaptive Therapeutics Animal Care and Use Committee (reference number: 2018010814547884-APAFIS#13479). To limit the bias induced by the natural murine coprophagia, mice were placed in a new cage on the day of administration of the bacterial suspension.

**FIGURE 1:**
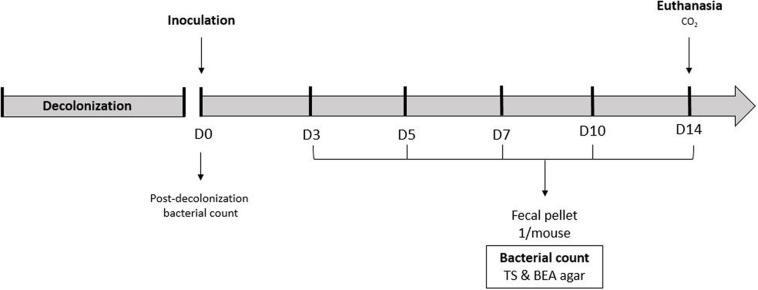
GIT colonization model used to compare antibiotic regimen.

To compare antibiotic regimen efficacy, mice received antibiotics to decolonize GIT according to the different protocols described below. At day 0, mice were orally inoculated, with the bacterial suspension of *E. faecium* WT. To avoid standard gavage, 2 days before D0, a “water + chocolate spread” suspension was orally administered with a syringe to mice for them to get used to it. On the day of inoculation, chocolate spread was added to the bacterial suspension at the very last moment and orally administered to each mouse. GIT colonization was evaluated by quantifying *E. faecium* in fresh fecal pellet (one per mouse) obtained by gentle abdominal massage and collected in sterile tubes. Fecal pellets were collected before inoculation (D0) to ensure decolonization, and at days 3, 5, 7, 10, and 14 (Figure 1). Samples were weighted and homogenized in 1 ml of saline solution. Mice were euthanized with CO_2_ at D14. Fecal pellets were quantitatively cultured onto TS and BEA agar plates for 18 h at 35 ± 2°C under ambient air, to quantify the aerobic flora and *E. faecium*, respectively. Bacterial loads were expressed in log_10_ cfu/g of stool or tissue for each sample.

Before the *ern0160* study, the *in vivo* plasmid stability was verified by performing the protocol with Δ0160_pAT29 strain on five mice. Fecal pellets were collected at D3 and D10 and plated on agar with and without spectinomycin. The stability was evaluated by the percentage of colony containing the pAT29 plasmid, calculated by the ratio$\frac{c\,f\,u\,\left(T\,S\,s\,p\,e\,c\right)}{c\,f\,u\,\left(T\,S\right)}$.

For the *ern0160* study, the same protocol was performed with 15 mice per group. Mice received antibiotics before bacterial suspension administration. The suspension contained each strain alone (WT, Δ0160, Δ0160_0160, or Δ0160_pAT29) or a two-strain combination prepared as described above (WT + Δ0160 or Δ0160_0160 + Δ0160_pAT29). A fecal pellet for each animal at D0, D3, D5, D7, and D10 were collected to be quantitatively cultured. Samples collected from mice colonized with WT or Δ0160 strains or the mixed suspension “WT + Δ0160” were cultured on TS and BEA agar. TS agar and TSspec agar were used to quantify bacterial load in samples collected from mice colonized with Δ0160_ 0160 or Δ0160_pAT29 strains and with the suspension “Δ0160_0160 + Δ0160_pAT29.” All agar plates were incubated for 18 h at 35 ± 2°C under ambient air. For co-colonization assay, each strain was identified by PCR, directly from colonies, under standard conditions and using specific primers (Supplementary Table 1). Twelve colonies were tested for each sample.

### Antibiotic Protocols

**FIGURE 2:**
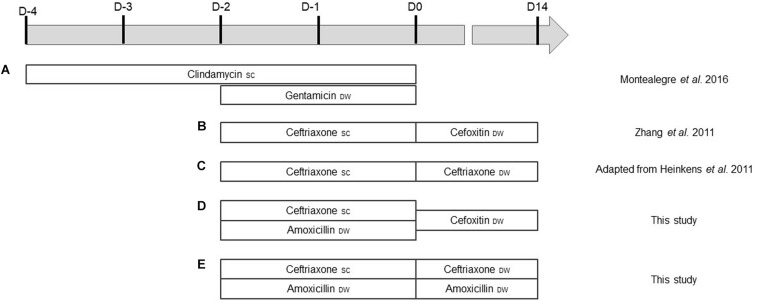
**(A–E)** Antibiotic administration according to the different protocols. D0 corresponds to the day of bacterial suspension inoculation. SC, subcutaneous; DW, drinking water.

Five decolonization regimens were tested. Each protocol was administered to five mice before the inoculation at D0. Protocol A, described by Montealegre et al. (2016), combined SC clindamycin (Panpharma S.A., Luitré, France) injected every 12 h (2.4 mg/day/mouse) for 4 days and gentamicin (Panpharma) in DW (1 mg/ml) for 2 days before inoculation. Zhang et al. (2011) described protocol B, which combined SC ceftriaxone (Mylan, Canonsburg, PA, United States) and cefoxitin (Panpharma). Ceftriaxone (2.4 mg/day/mouse) was injected twice a day, 2 days before inoculation and cefoxitin (0.2 mg/ml) added to DW during the 14 days of the colonization. Protocol C was adapted from Heikens et al. (2009), who used SC ceftriaxone (2.4 mg/day/mouse) 2 days before inoculation and until the end of the study. In our adapted protocol, SC ceftriaxone was administered (2.4 mg/day/mouse) 2 days before inoculation and then in DW (0.1 mg/ml) until D14. Protocol D was the same as protocol B (ceftriaxone plus cefoxitin), with amoxicillin (Panpharma) (0.1 mg/ml) added in DW during the 2 days before inoculation. Finally, protocol E combined SC ceftriaxone (2.4 mg/day/mouse) and amoxicillin in DW (0.1 mg/mL) 2 days before inoculation; ceftriaxone (0.1 mg/ml) and amoxicillin (0.03 mg/ml) were then put in DW throughout the colonization. All regimens are summarized in Figure 2. One group received no antibiotics as a control group.

### Statistical Analysis

For competitive and co-colonization assays, competitive index (CI) and normalized competitive index (nCI) were calculated for each time point as follows. CI was the ratio between “Δ0160 colonies” and “WT colonies,” or between “Δ0160_0160 colonies” and “Δ0160_pAT29 colonies.” nCI was calculated by dividing CI by CI calculated at T0 for *in vitro* assay, and D0 (suspension inoculated) for *in vivo* experiments. A normalized competitive index of 1 meant no difference, a normalized competitive index < 1 meant that WT or Δ0160_pAT29 was in higher numbers than Δ0160 or Δ0160_0160, respectively, and a competitive index > 1 meant that Δ0160 or Δ0160_0160 was in higher numbers than WT or Δ0160_pAT29, respectively. A Shapiro–Wilk test (*p* value >0.05) was used to evaluate normality. According to the result, CIs were analyzed using Student’s *t* test or Wilcoxon signed-rank test, with the null hypothesis: mean CI was not significantly different from 1 (*p* values of 0.05 used). Between-group cfu counts for fecal pellets were expressed by mean and standard deviation and compared with the Mann–Whitney test or Student’s *t* test. Antibiotic protocol results were analyzed using a Kruskal–Wallis test. *p* < 0.05 was considered statistically significant. Data were analyzed using GraphPad Prism 7 (GraphPad Software, Inc.).

## Results

### *In vitro* Experiments

**FIGURE 3:**
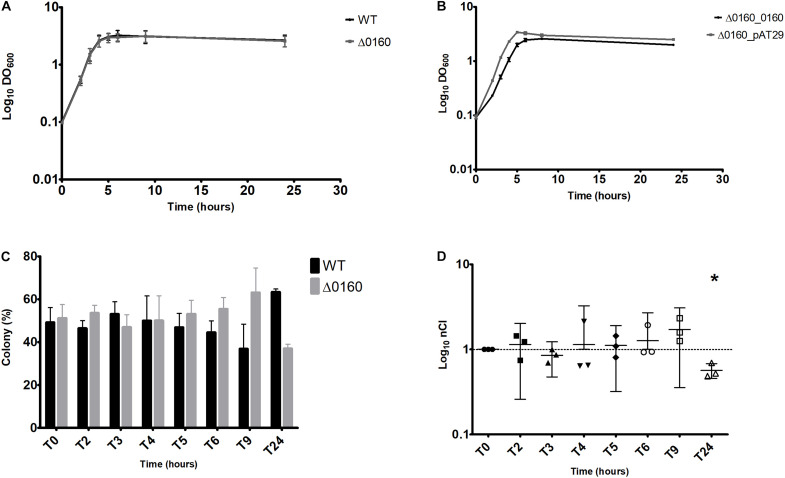
*In vitro* experiments. **(A)** Bacterial growth curves of WT and Δ0160 strains growth individually in BHI broth. **(B)** Bacterial growth curves of Δ0160_0160 and Δ0160_pAT29 strains growth individually in BHI broth supplemented with spectinomycin. Each mean value in panels **(A)** and **(B)** represents the mean DO_600_ generated from three replicates; error bars represent standard error. **(C)** Competitive assay of WT *versus* Δ0160 strains. Proportion of colonies of each strain identified by PCR colony. Each mean value represents the mean colony proportion from three replicates (28 PCR at each time point for each replicate); error bars represent standard error. **(D)** Competitive assay of WT versus Δ0160 strains; normalized competitive index (nCI) values of each replicate represented with means and 95% confidence interval. Asterisk indicates which results have been shown by statistical analysis to be different from 1.

Results for all bacterial growth curves are represented in Figures 3A,B. No significant difference was observed between WT and Δ0160 growth curves. The maximum DO_600_ was similar and achieved simultaneously. Conversely, Δ0160_0160 and Δ0160_pAT29 showed a different growth rate. A difference of approximately 2 h to reach the growth plateau was observed between the two strains, and Δ0160_0160 grew more slowly than Δ0160_pAT29. Moreover, the maximum DO_600_ was lower for Δ0160_0160 bacterial growth.

According to these results, only the WT and Δ0160 strains were studied in competitive assays. A competitive assay was conducted during 24 h with PCR on 28 different colonies performed at each point of time. Results are reported in Figures 3C,D. Up to 9 h of culture, the nCI was not significantly different from 1, which means that the two strains were growing in a similar way. At T24, the mean nCI was 0.57 and significantly lower than 1 (*p* = 0.02), meaning that the WT strain was predominant compared to the deleted strain Δ0160.

### *In vivo* Experiments

#### Experimental Model Development

Each protocol was tested on five mice and colonized with the *E. faecium* WT suspension for 14 days. Prior to beginning of our experiments, normal aerobic GIT microbiota of Swiss mice was determined on five mice. It was composed of lactobacilli (85.2%), Enterobacterales (8.5%), and *E. faecalis* (6.3%). For each experiment, mice were screened for *E. faecium* before colonization (D0) and no stool samples contained any.

**FIGURE 4:**
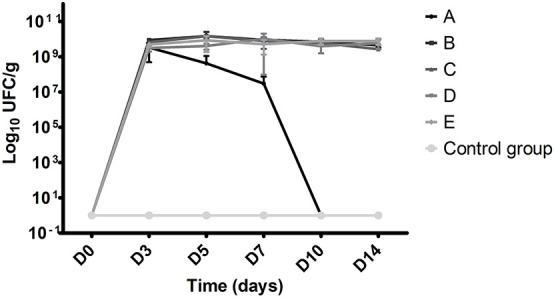
VREF bacterial load in fecal pellets according to antibiotic regimen and time. Each point represents the mean value of cfu values per gram obtained from five mice. A Clindamycin SC + gentamicin DW, B ceftriaxone SC + cefoxitin DW, C ceftriaxone SC + DW, D ceftriaxone SC + amoxicillin DW + cefoxitin DW, and E ceftriaxone + amoxicillin SC and DW (SC, subcutaneous; DW, drinking water).

Results are shown in Figure 4. In the control group, which received no antibiotic prior to WT suspension administration, no VREF were isolated from fecal pellets at D3, 5, 7, 10, and 14. With protocol A (gentamicin + clindamycin), fecal pellets contained a mean of 3.34 × 10^9^ cfu/g at D3, 4.24 × 10^8^ cfu/g at D5, and 3.00 × 10^7^ cfu/g at D7. No VREF were found between D10 and D14. Whereas there were no *E. faecalis* at D0, they were gradually detected from D7 to D14 (3.08 × 10^4^ cfu/g at D14). With protocols B (ceftriaxone + cefoxitin), C (ceftriaxone alone), D (ceftriaxone + cefoxitin + amoxicillin), and E (ceftriaxone + amoxicillin), a high and stable colonization was observed. Between D3 and D14, fecal pellets from mice treated with protocols B and C contained a mean of 8.86 × 10^9^ cfu/g and 5.50 × 10^9^ cfu/g, respectively. Some *E. faecalis* were detected at the end of colonization, but they remained a minority compared to VREF. The mean VREF bacterial load in fecal pellets from animals receiving protocols D and E antibiotics were 8.89 × 10^9^ and 6.69 × 10^9^ cfu/g, respectively. No contamination by *E. faecalis* was observed with protocol E. Regarding VREF colonization, no significant difference was observed between protocols B, C, D, and E (*p* = 0.6).

#### Ern0160 Study

To study *in vivo* the role of Ern0160 in GIT colonization, the model with protocol E (ceftriaxone plus amoxicilline) was applied during 10 days, with each strain alone in a first step (WT, Δ0160, Δ0160_0160, or Δ0160_pAT29) and with a combination of two in a second step (WT + Δ0160 and Δ0160_0160 + Δ0160_pAT29). Each group was composed of 15 mice. The *in vivo* stability was first evaluated by determining the percentage of colony that retained the pAT29 plasmid. At D3, 94% of colonies retained the plasmid and 75% at D10, which is consistent with studies previously published (Deol et al., 2007).

**FIGURE 5:**
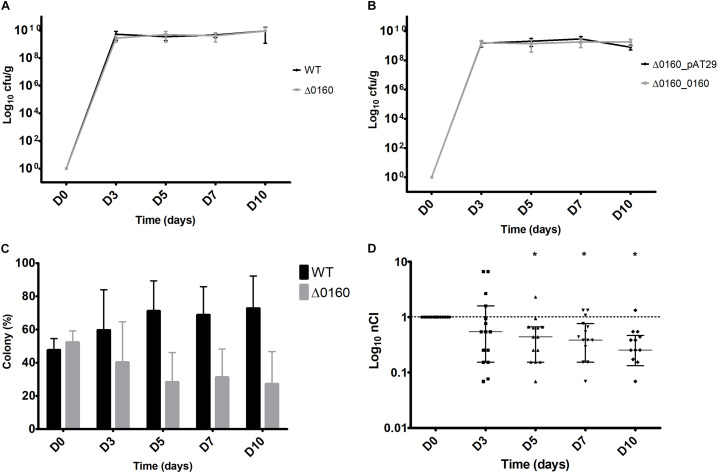
GIT colonization with each strain individually and co-colonization with WT and Δ0160 strains mixed. **(A)** Mean bacterial load per gram in fecal pellets from 15 mice inoculated with the WT strain, and 15 mice with the Δ0160 strain. **(B)** Mean bacterial load per gram in fecal pellets from mice inoculated with mutant strains Δ0160_0160 and Δ0160_pAT29. Error bars represent standard deviation. **(C)** Proportion of colonies of each strain identified by PCR colony. Each mean value represents the mean colony proportion from 15 mice (12 PCR at each time point for each sample). **(D)** Normalized competitive index (nCI) values calculated for each mouse represented with means and 95% confidence interval. Asterisk indicates which results have been shown by statistical analysis to be different from 1.

Results obtained with GIT colonization with single strains are shown in Figures 5A,B. At D3, the mean bacterial load was significantly higher with the WT strain compared to Δ0160 (5.2 × 10^9^ cfu/g and 2.8 × 10^9^ cfu/g, respectively, *p* = 0.008). Then, from D5 to D10, GIT colonization remained high and stable with WT and Δ0160 strains, with a mean bacterial load of 5.78 × 10^9^ and 6.03 × 10^9^ cfu/g, respectively, which was not significantly different (Figure 5A). A high and stable GIT colonization was also obtained from D3 with mutant strains Δ0160_0160 and Δ0160_pAT29. At D3, the mean bacterial load was 1.49 × 10^9^ and 1.61 × 10^9^ cfu/g with Δ0160_0160 and Δ0160_pAT29, respectively, which was not significantly different. From D5 to D10, GIT colonization remained stably high with Δ0160_0160 and Δ0160_pAT29 strains, with a mean bacterial load of 5.93 × 10^9^ and 1.70 × 10^9^ cfu/g, respectively, which was not significantly different (Figure 5B).

The co-colonization model was performed with the two combinations of strains. With WT + Δ0160 suspension, the nCI was not significantly different from 1 at D3; the two strains colonized GIT in the same way. From D5 to D10, nCI significantly decreased (*p* = 0.01 at D5, and *p* < 0.01 at D7 and D10) with a predominance of the WT strain (Figures 5C,D). With Δ0160_0160 + Δ0160_pAT29 suspension, an nCI under 0.1 was observed from D3 to the end of the protocol for all mice. At D10, all colonies tested belonged to the Δ0160_pAT29 strain and no Δ0160_0160 colony was isolated.

## Discussion

In the last decade, bacterial sRNAs have been more and more investigated, and even if functions of many of these riboregulators are still unknown, several of them are involved in stress response or virulence. Few data are available on sRNAs in *E. faecium*; indeed, they have been identified only recently (Sinel et al., 2017). In their study, Sinel et al. showed that the expression level of one sRNA, Ern0160, was significantly downregulated under SIC daptomycin exposure. More recently, Dejoies et al. (2021) described a significant reduction in *ern0160* expression level under SICs of biocides. These two studies suggest that Ern0160 could be involved in antibiotic and biocide stress responses in *E. faecium*, and for these reasons, it appeared to be an interesting candidate potentially involved in its pathogenicity. GIT colonization is the main starting point of VREF infection and hospital outbreaks. Colonization mechanisms remain unclear, so this study sought to highlight whether Ern0160 could be implicated.

GIT colonization studies are difficult to reproduce *in vitro* because many parameters are involved, such as pH, cell diversity, or enzymatic activity. Experimental models have been developed to study enterococcus infectious diseases since 1899 (Maccallum and Hastings, 1899). These models enabled researchers to study enterococcal pathogenesis in the context of the innate and adaptive immune responses, as closely as possible to human conditions. Moreover, experimental models are reproducible and reliable statistical analysis on data could be done. Mice are the most frequently used animal to study enterococci in GIT (Goh et al., 2017). In our study, we evaluated for the first time sRNA of *E. faecium* in a GIT colonization model optimized for this purpose. Several models of GIT colonization have been published, based on the administration of a suspension of enterococci *via* oral gavage or in the drinking water. Oral gavage is an invasive procedure that can damage the esophagus, but a calibrated quantity of bacteria could be administered. Because mice are housed by five, if the bacterial suspension is administered in the drinking water, the number of bacteria taken up by each mouse is not precisely quantifiable. Because of these disadvantages, an alternative method has been chosen for our study (Scarborough et al., 2020). A “water+chocolate spread” solution was orally administered to mice a few days before starting colonization and then added to bacterial suspension. Thus, a calibrated suspension of bacteria could be administered without invasive procedure and thus avoid the possible associated complications. Prior to inoculation, mice received antibiotics to eliminate GIT normal flora. Several antibiotic regimens have been published and compared in our study. All tested protocols led to an important colonization but had some limitations. With the one associating clindamycin and gentamicin, the VREF colonization did not persist long enough and with protocols composed of β-lactams without amoxicillin, a significant growth of *E. faecalis* was observed. A high and stable colonization without *E. faecalis* contamination was obtained with two regimens, ceftriaxone associated with amoxicillin and ceftriaxone associated with amoxicillin and cefoxitin. As cefoxitin is not frequently used in human medicine unlike ceftriaxone, the protocol without cefoxitin was preferred, to be as close as possible to human conditions.

In our *in vitro* experiments, the deleted mutant strain Δ0160 grew like the wild-type strain. These results have been confirmed in *in vivo* experiments. Indeed, a similar colonization profile was obtained with the two strains tested individually. Competitive assays have also been performed *in vitro* and *in vivo*. *In vitro*, neither of the two strains grew more than the other up to 24 h of culture. At 24 h, the wild-type strain was slightly predominant. As *in vitro* experiments were performed in BHI broth, which is far from the composition of the digestive tract, it was important to perform competition assays in mice. In the co-colonization model, the wild-type strain was significantly predominant from day 3 until the end of the protocol. These results suggested that Ern0160 could be involved in the GIT colonization process. To complete the results obtained with the deleted strain, identical *in vitro* and *in vivo* experiments were performed with the *trans*-complemented strain Δ0160_0160, which overexpressed *ern0160*, knowing that overexpression experiments are classically used to study sRNA functions (Brantl and Jahn, 2015). The strain Δ0160_pAT29, with an empty pAT29 vector, was used as control to compare strains with supposed similar fitness costs. In individual bacterial growth curve assays, the Δ0160_0160 strain appeared to grow more slowly than the Δ0160_pAT29 strain. The overexpression of *ern0160* by the strain Δ0160_0160 may induce an energetic cost for the bacterium and could therefore explain the shift observed in growth kinetics. In the colonization model with only a strain, the two strains colonized mice in the same way during all protocol. In the co-colonization model, the Δ0160_pAT29 strain was largely predominant by day 3 and until the end of the protocol. Based on our first results, it would have been expected that the complemented strain Δ0160_0160 would colonize GIT more than the Δ0160_pAT29 strain. As the Δ0160_0160 strain overexpressed *ern0160* compared to the WT strain, these results suggested that an overexpression of *ern0160* could be deleterious to the strain in GIT colonization. Altogether, these findings show the involvement of Ern0160 in GIT colonization with a probable very tight regulation. Further studies, in particular with a *cis*-complemented strain, may provide additional information. As Ern0160 has probably pleiotropic effects, it will also be important to determine which targets are regulated by Ern0160 to understand precisely molecular mechanisms. As the different pathways of adaptation or virulence often involve several RNAs, it will also be interesting to determine if other sRNAs are involved in GIT colonization and if there is a cross-regulation with Ern0160. This study was the first to explore *in vivo E. faecium* regulatory RNAs and its potential function in GIT colonization. Further studies are needed to determine the targets of this sRNA in order to decipher its role and regulatory circuits.

## Data Availability Statement

The raw data supporting the conclusions of this article will be made available by the authors, without undue reservation.

## Ethics Statement

The animal study was reviewed and approved by Adaptive Therapeutics Animal Care and Use Committee.

## Author Contributions

SR, BF, VC, and MR conceived and designed the experiments, analyzed the data, and wrote the manuscript. SR, KL, VB, LD, and AL performed the experiments. VB, BF, VC, and MR contributed with reagents, materials, and analysis tools. All the authors discussed the results and commented on the manuscript.

## Conflict of Interest

The authors declare that the research was conducted in the absence of any commercial or financial relationships that could be construed as a potential conflict of interest.

## Publisher’s Note

All claims expressed in this article are solely those of the authors and do not necessarily represent those of their affiliated organizations, or those of the publisher, the editors and the reviewers. Any product that may be evaluated in this article, or claim that may be made by its manufacturer, is not guaranteed or endorsed by the publisher.
