# Supplementary material for: The Regulatory RNA ern0160 Confers a Potential Selective Advantage to Enterococcus faecium for Intestinal Colonization
Source: Front Microbiol. 2021 Nov 10;12:757227. doi: 10.3389/fmicb.2021.757227 (PMC8631354; doi:10.3389/fmicb.2021.757227)
Supplement: Supplementary file 1 [file Table_1.docx]

**Supplementary Table S1 |** Oligonucleotides used in the study.

| Name | Nucleotide sequence (5’-3’) | Gene and purpose |
| --- | --- | --- |
| KOern0160 F1 | TTGACCCGGGCTATCTGACACTACATGTAG | *ern0160* deletion and verification |
| KOern0160F2 | CTCTCGATAAGCTCGATCTATATAATAGCATCCTTTG |  |
| KOern0160R1 | CGAGCTTATCGAGAGCTCGGCTTTTTC |  |
| KOern0160R2 | ACACCCGGGGTTTGTTTATTCATATCTTGC |  |
| KOern0160VF | GTGCTTATATTTGCAAACGCTACCAAAGG |  |
| KOern0160VR | GGAGATGCGATGTTCGTGAAATAAGG |  |
| pCern0160F2 | GGTGGTGGATCCGGTTATGAAAAGCCGCTAG |  |
| pCern0160R2 | GGTGGTGAATTCCCTATTCCTCCTTTAAAATCTTCGTAC |  |
| pWS3F1 | TTTCCCAGTCACGACGTTGT | Plasmid verification |
| pWS3R1 | ACTGACAGCTTCCAAGGAGC |  |
| pAT29vF | GTAAAACGACGGCCAG |  |
| pAT29vR | CAGGAAACAGCTATGAC |  |
